# Finished Whole-Genome Sequence of *Clostridium argentinense* Producing Botulinum Neurotoxin Type G

**DOI:** 10.1128/genomeA.00380-17

**Published:** 2017-05-25

**Authors:** Jessica L. Halpin, Karen Hill, Shannon L. Johnson, David Carlton Bruce, T. Brian Shirey, Janet K. Dykes, Carolina Lúquez

**Affiliations:** a Centers for Disease Control and Prevention, Atlanta, Georgia, USA; b Los Alamos National Laboratory, Los Alamos, New Mexico, USA

## Abstract

Here, we present a closed genome sequence for *Clostridium argentinense* strain 89G, the first strain identified to produce botulinum neurotoxin type G (BoNT/G). Although discovered in 1970, to date, there have been no reference quality sequences publicly available for this species.

## GENOME ANNOUNCEMENT

Botulism is a rare but serious neuroparalytic disease that is caused by botulinum neurotoxins. These toxins are produced by *Clostridium botulinum* and several other *Clostridia* species, among them *Clostridium argentinense* (1). *C. argentinense* producing botulinum toxin type G was originally isolated from soil samples in the province of Mendoza, Argentina, in the 1960s (2–4). To date, no humans have been treated for botulism due to toxin type G, but it is essential to have the ability to quickly identify any such cases in the future, and this sequence will aid in this effort (5).

A hybrid assembly method was utilized, and the isolate was sequenced by Illumina and PacBio and then assembled using Velvet 1.2.08, HGAP 3, and Phrap 4.24. Average coverage depths of 152× by Illumina and 328× by PacBio were achieved.

The genome revealed a plasmid, pRSJ17_1 (accession no. CP014175), of 140,070 bp that consists of 26.7% G+C content and encodes 129 genes. The plasmid includes no coding regions for tRNA or rRNA but does include the 3,937-bp gene that encodes the botulinum neurotoxin. The chromosome has a length of 4,662,988 bp, consisting of 28.8% G+C content. The chromosome (accession no. CP014176) includes 4,145 open reading frames and 95 tRNA- and 30 rRNA-coding regions.

### Accession number(s).

This sequence has been deposited in GenBank under the accession numbers CP014175 (plasmid) and CP014176 (chromosome).
